# PREVENTABLE HOSPITALIZATIONS IN ADULTS WITH ALZHEIMER’S DISEASE AND RELATED DEMENTIAS, 2016–2019

**DOI:** 10.1093/geroni/igae098.2594

**Published:** 2024-12-31

**Authors:** Jenny Walker, Roshni Patel, Benjamin Olivari, Christopher Taylor, Matthew Baumgart, Lisa McGuire

**Affiliations:** Centers for Disease Control and Prevention, Atlanta, Georgia, United States; Cyberdata Technologies (CDC Contractor), Atlanta, Georgia, United States; Centers for Disease Control and Prevention, Atlanta, Georgia, United States; Centers for Disease Control and Prevention, Atlanta, Georgia, United States; Alzheimer’s Association, Chicago, Illinois, United States; GSA, Atlanta, Georgia, United States

## Abstract

Reducing preventable hospitalizations in adults with Alzheimer’s disease and related dementias (ADRD) is a public health and National Healthy People 2023 priority because the associated negative health and financial outcomes may be avoidable through proactive primary and outpatient care recognizing and managing co-morbidities. This study used data from the 2016–2019 Healthcare Cost Utilization Project National Inpatient Sample, an all-payer inpatient care database. Using ICD-10-CM codes, we identified 8 conditions that are potentially preventable, including acute and chronic ambulatory care sensitive conditions and injuries. Of the hospital discharges among adults aged ≥45 years with ≥1 of 11 conditions (n=4,611,423), 11.2% (95%CI=11.1-11.3) also listed an accompanying ADRD diagnosis for the patient. Among adults with an ADRD diagnosis, the proportion of potentially preventable hospitalizations increased linearly with age, with 82.4% (95%CI=82.2-82.5) of potentially preventable hospitalizations occurring among those aged ≥75 years. In contrast, potentially preventable hospitalizations among those without ADRD skewed younger, with only 34.2% (95%CI=34.0-34.4) occurring among those aged ≥75 years. Of the 8 potentially preventable conditions, most common among those with ADRD were sepsis (34.1%), injuries (21.1%), urinary tract infections (UTIs; 14.0%), and heart failure (12.9%). Adults with ADRD were more likely to be hospitalized due to UTIs than those without ADRD (adjusted prevalence ratio=2.34 (95%CI=2.32-2.37)), even after adjusting for sex, age, and long-term care facility residency. Future public health measures, policy, and programs with targeted ADRD hospitalization prevention goals may potentially mitigate the issue, with special attention to increased risk of hospitalization due to older age and UTIs.

